# Free Charge Carrier Generation by Visible-Light-Absorbing Organic Spacers in Ruddlesden–Popper Layered Perovskites

**DOI:** 10.1021/jacs.4c09706

**Published:** 2024-09-24

**Authors:** Simon Nussbaum, Demetra Tsokkou, Aaron T. Frei, Dennis Friedrich, Jacques-E. Moser, Natalie Banerji, Jun-Ho Yum, Kevin Sivula

**Affiliations:** † Laboratory for Molecular Engineering of Optoelectronic Nanomaterials, Institute of Chemical Sciences and Engineering (ISIC), École Polytechnique Fédérale de Lausanne (EPFL), 1015 Lausanne, Switzerland; ‡ FemtoMat Research Group, Department für Chemie, Biochemie und Pharmazie, University of Bern, Freiestrasse 3, 3012 Bern, Switzerland; § Photochemical Dynamics Group, Institute of Chemical Sciences and Engineering (ISIC), École Polytechnique Fédérale de Lausanne (EPFL), 1015 Lausanne, Switzerland; ∥ Institute for Solar Fuels, Helmholtz Zentrum Berlin für Materialien und Energie, Hahn-Meitner-Platz 1, 140109 Berlin, Germany

## Abstract

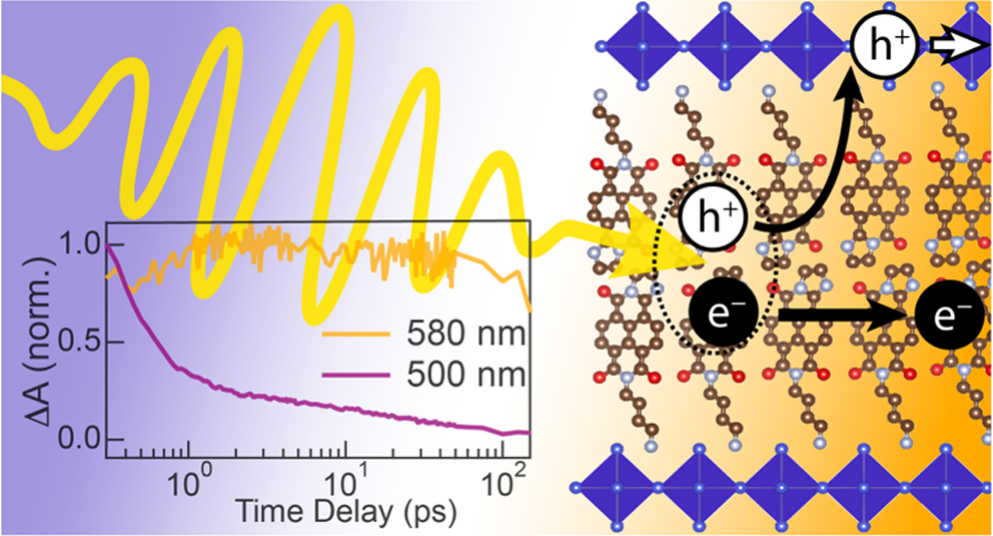

Incorporating organic semiconductor building blocks as spacer cations into layered hybrid perovskites provides an opportunity to develop new materials with novel optoelectronic properties, including nanoheterojunctions that afford spatial separation of electron and hole transport. However, identifying organics with suitable structure and electronic energy levels to selectively absorb visible light has been a challenge in the field. In this work, we introduce a new lead-halide-based Ruddlesden–Popper perovskite structure based on a visible-light-absorbing naphthalene-iminoimide cation (NDI-DAE). Thin films of (NDI-DAE)_2_PbI_4_ show a quenched photoluminescence and transient absorption dynamics consistent with the formation of a charge transfer state or free charge carriers when either the inorganic or organic layer is photoexcited, suggesting the formation of a type II nanoheterostructure. Time-resolved microwave conductivity analysis supports free charge generation with sum mobilities up to 4 × 10^–4^ cm^2^ V^–1^ s^–1^. Mixed halide (NDI-DAE)_2_Pb(I_*x*_Br_1–*x*_)_4_ films show modified inorganic layer band gaps and a photoluminescent reversed type I nanoheterostructure with high bromide content (e.g., for *x* = 0). At *x* = 0.5, transient absorption and microwave conductivity measurements provide strong evidence that selective visible-light absorbance by the NDI-DAE cation generates separated free carriers via hole transfer to the inorganic layer (leaving photogenerated electrons in the organic layer), which represents an important step toward enhancing light harvesting and affording the spatial separation of charge carrier transport in stable layered perovskite-based devices.

## Introduction

Layered hybrid organic–inorganic perovskites (LHOIPs), often called “2D” perovskites, are a novel class of semiconductors that have attracted significant recent interest for application in various optoelectronic devices such as light-emitting diodes,^1−3^ solar cells,^4−6^ field effect transistors (FET),^7,8^ and X-ray detectors.^9,10^ Conceptually, LHOIPs are formed by replacing the A-site cation in a conventional “3D” perovskite with the general formula ABX_3_ with a large, bulky organic spacer cation. As these spacer cations are too large to fit into the perovskite framework, a crystal structure of alternating organic/inorganic layers forms along a particular crystallographic direction.^11^ In the most commonly investigated case where aliphatic cations such as butylammonium (BA) or small aromatics such as phenylethylammonium (PEA) are incorporated into lead halide perovskites, the spacer acts primarily structurally—separating inorganic slabs of corner-sharing PbX_6_ octahedra.^12^ Due to the mismatch between the electron energies of the lowest unoccupied molecular orbital (LUMO) and the highest occupied molecular orbital (HOMO) of the organic spacer with the conduction band (CB) and valence band (VB) of the inorganic layer, a quantum well electronic structure is formed, where excited electrons and holes are localized solely in the inorganic perovskite slabs. The resulting electronic and dielectric confinement in these materials generates highly anisotropic charge carrier transport and distinct optoelectronic properties dominated by high exciton binding energies.^13−15^ Recent research has focused on functionalizing the spacer cation to tune the optoelectronic properties of the LHOIPs and to overcome potential drawbacks for application in optoelectronic devices.^16,17^ In this context, an interesting approach is the incorporation of pi-conjugated organic semiconducting moieties as spacer cations.^18−22^ Unlike aliphatic spacer cations, organic semiconducting spacers can have HOMO/LUMO levels suitable for electronic interaction with the inorganic layer,^23,24^ forming, for example, type II nanoheterostructure systems^22^ that afford the localization of photogenerated electrons and holes in separate layers at the nanometer scale. Previous work has suggested that the resulting long-lived charge transfer (CT) excitons in type II nanoheterostructures may boost the charge separation and free charge carrier generation.^25,26^ While the effect of the electronic interactions between the organic layer and the inorganic layer has been demonstrated by our group and others,^27−29^ and colloidal perovskite nanoparticle systems coated with organic chromophores have been studied as model systems,^30−34^ given the overlap of light absorption of the inorganic and organic components in Pb-based LHOIPs, it remains unclear how photon absorption by the organic layer can contribute to the light harvesting and free charge generation processes. However, simply increasing the size of the pi-conjugated organic in order to decrease its HOMO/LUMO gap is problematic as the incorporation of increasingly bulky cations into an LHOIP is limited. In this work, we prepare a novel visible-light-absorbing spacer cation based on a naphthalene-iminoimide core and, upon incorporating it into a layered perovskite structure, demonstrate that the photon absorption in the spacer cation can effectively contribute to the free charge carrier formation in LHOIPs.

## Results and Discussion

**Figure 1:**
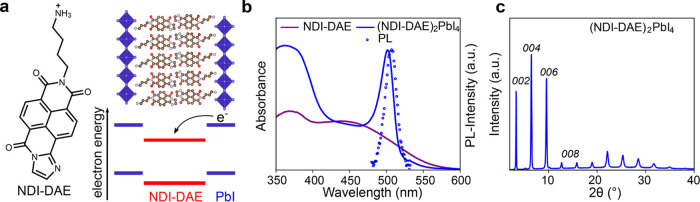
(a) Chemical structure of the NDI-DAE spacer cation and illustration of the aimed layered perovskite structure. (b) Visible-light absorbance of NDI-DAE cation (violet solid line) and (NDI-DAE)_2_PbI_4_ (blue solid line), and PL emission (excited at 400 nm, blue dotted line). (c) XRD of (NDI-DAE)_2_PbI_4_ layered perovskite thin film.

In order to identify an organic spacer cation capable of incorporating into a LHOIP while also providing a low excitation energy for selective light absorption, we turned to rylene-based compounds, which are a widely studied class of organic semiconductors that offer many possibilities for electronic structure modulation. Functionalization at either the longitudinal diimide position or the lateral core position can effectively alter the HOMO/LUMO gap; however, since we aim to keep the width of the chromophore small enough for incorporation into a LHOIP, we chose to extend the aromatic system of the NDI-core at the diimide position. The condensation of naphthalenetetracarboxylic dianhydride subsequently with one equivalent of 1,2-diaminoethane^35^ and then *N*-Boc-1,4-diaminobutane followed by acid hydrolysis (as described in the Supporting Information, Scheme S1) afforded a novel cation, coded as NDI-DAE (see structure in Figure 1a, and basic characterization in the Supporting Information). The NDI-DAE cation was incorporated into a Ruddlesden–Popper LHOIP structure, (NDI-DAE)_2_PbI_4_ (shown schematically in Figure 1a), as thin films prepared by spin-coating a precursor solution containing PbI_2_ and the NDI-DAE iodide salt (1:2 stoichiometric ratio) in dimethyl sulfoxide (DMSO) on SiO_2_ substrates and subsequently annealing at 200 °C. As discussed in previous work,^27^ the high-temperature annealing was found to be a crucial parameter for the formation of the layered perovskite phase, compared to typical LHOIP materials such as (BA)_2_PbI_4_, as shown by in situ temperature-dependent UV–vis spectroscopy (Figure S1). The UV–vis absorption of the resulting (NDI-DAE)_2_PbI_4_ film is shown compared to that of a thin film of pure NDI-DAE in Figure 1b. While the pure NDI-DAE salt showed a broad absorption in the range between 350 and 560 nm, an additional sharp excitonic absorption band at around 510 nm appears in the (NDI-DAE)_2_PbI_4_ thin film. Moreover, a photoluminescence (PL) emission at about 520 nm (excitation at 400 nm) was observed (Figure 1b, data points). Such a characteristic excitonic absorption band with a sharp PL is similar to what is frequently reported in the Ruddlesden–Popper phase incorporating BA or PEA, suggesting the formation of a layered RP-phase structure.^11,36^

The formation of a crystalline and in-plane-oriented layered phase is further supported by X-ray diffraction (XRD) and grazing-incident wide-angle X-ray scattering (GIWAXS) data, as shown in Figures 1c and S2, respectively, where strong and periodic diffraction peaks associated with the 00-planes can be observed. Based on the 002-diffraction peak, an interplanar distance of 27 Å is estimated, which corresponds to approximately twice the length of the spacer cation and is comparable with the interplanar distance of other NDI-based RP LHOIPs.^27,28^ While the small Stokes shift in the PL is a characteristic of the RP phase, it should be noted that the PL intensity was strongly reduced in (NDI-DAE)_2_PbI_4_ compared to that in (BA)_2_PbI_4_, as displayed in Figure S3. Based on the electron-accepting properties of such rylene dyes, the PL quenching could be ascribed to the formation of a type II nanoheterostructure, where photoexcited electrons in the inorganic layers transfer to the NDI-DAE at a time scale faster than recombination. Ultraviolet photoelectron spectroscopy (UPS) data of NDI-DAE (Figure S4), compared to UPS data of (BA)_2_PbI_4_ (vide infra), support this possibility by confirming the electronic energy level alignment, which indicates that the HOMO energy level of the NDI-DAE lies below the valence band (VB) of the inorganic Pb–I layer as shown schematically in Figure 1a.

**Figure 2:**
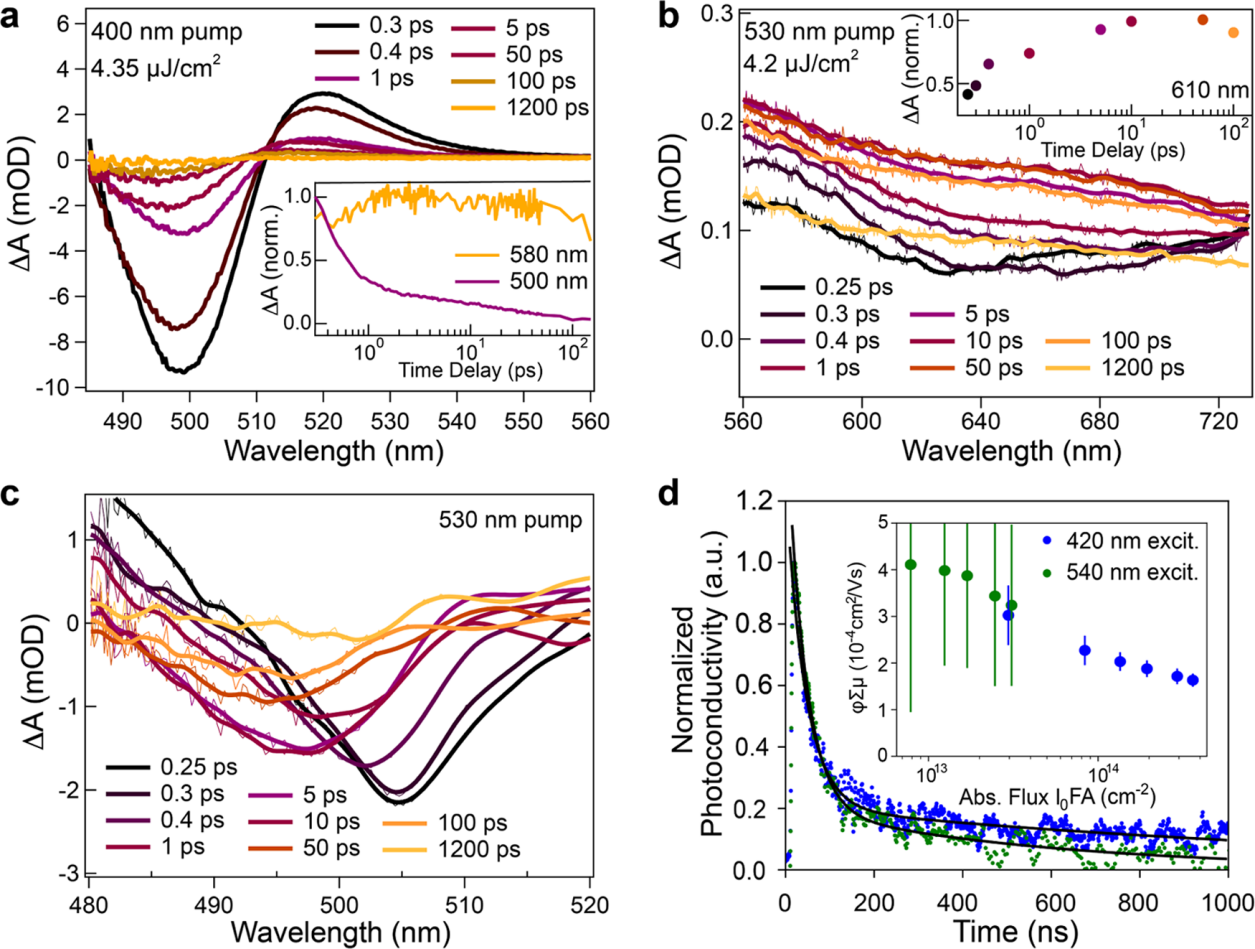
(a) Transient absorption spectra of (NDI-DAE)_2_PbI_4_ films and GSB dynamics (inset) after excitation at 400 nm and (b) after excitation at 530 nm. (c) The selective excitation of the spacer chromophore leads to a ground state bleach below 500 nm, suggesting hole injection into the inorganic layer. (d) fp-TRMC transient and peak photoconductivity after excitation at 420 and 540 nm (inset).

To confirm that the observed PL quenching is due to photoexcited charge transfer and to further investigate the charge transfer dynamics, we turned to femtosecond transient absorption (TA) spectroscopy measurements. Thin films were excited with a 70 fs laser pulse, and the change in the optical absorbance was monitored by a broadband probe pulse. A low pump fluence of ∼4 μJ/cm^2^ at room temperature was used to minimize exciton–exciton interactions due to multiple exciton generation. The absorbance change spectra at various delay times after photoexcitation at 400 nm are displayed in Figure 2a and show the expected features: a ground state bleach (GSB) centered around 500 nm and photoinduced absorption (PIA) peaking at about 520 nm. The PIA can be associated with band gap renormalization,^37^ while the GSB is most reasonably ascribed to phase space filling after depopulation of the ground state of the material.^38^ The GSB minimum shows only a minor shift after a few picoseconds, suggesting the formation of only single excitons. The dynamics of the GSB (purple solid line) are displayed in the inset of Figure 2a and show a fast decay with an initial time constant of about 1–2 ps, which matches the rise of an additional broad PIA band seen at wavelengths longer than 550 nm. The dynamics of this PIA at 580 nm are shown in the inset of Figure 2a (orange line), and the absorbance change spectra for the 560–720 nm range are shown in Figure S5. Importantly, this broad PIA band, which peaks at around 650 nm, is different in shape and shows the evolution in different time scales when films of pure NDI-DAE salt are excited at 400 or 530 nm, as shown in Figure S6. Thus, we believe that this PIA band is related to electron transfer from the photoexcited PbI_4_ slabs to the NDI-DAE and that this process occurs within a few ps. We note that the charge transfer time scale observed here is comparable to the PL decay dynamics in similar NDI-based RP perovskites.^27^

However, unlike other LHOIP systems, the organic layer in (NDI-DAE)_2_PbI_4_ can absorb photons with energy lower than that of the primary excitonic transition of the inorganic layers (510 nm), which offers an additional wavelength region to probe this material. When (NDI-DAE)_2_PbI_4_ films were excited at 530 nm, the broad PIA feature in the range between 560 and 700 nm was also observed, as displayed in Figure 2b. Notably, the signal monitored at 580 nm rises with a similar initial time constant as the GSB of the perovskite, which is observed at around 500 nm when using the 530 nm pump (Figure 2c), analogous to what is observed with 400 nm excitation. However, with 530 nm excitation, the rise of the PIA band is slower compared to the case when using the 400 nm pump, peaking at 10 ps instead of about 2 ps (see Figure 2b, Inset). Therefore, the PIA from the 530 pump can be attributed to a different charge transfer process. Reasonably, due to the larger fraction of 530 nm photons being directly absorbed by the organic, the rise of the PIA is ascribed to photogenerated hole transfer from the photoexcited NDI-DAE to the perovskite. This is consistent with the established electronic structure, where the HOMO level of NDI-DAE is located energetically below the VB of the inorganic layer. Thus, hole injection from the photoexcited organic layer to the inorganic layer would be expected, as previously suggested in colloidal perovskite systems with organic dye ligands,^25,26,33^ Accordingly, the GSB at ca. 500 nm when using the 530 nm pump (Figure 2c) can be ascribed to depopulation of the VB electrons in the inorganic layer by hole injection. When comparing the perovskite GSB with 530 and 400 nm excitation, a small blue shift is seen (500 nm vs 495 nm upon, respectively). While we are unable to unambiguously determine the cause, we note that a photoinduced Stark effect due to the electric field induced by bound excitons or charges at the organic–inorganic interface may explain the small change in the energy of this state.^39^

Regarding the broad PIA in the range between 560 and 700 nm, which is produced under both 400 and 530 nm pump conditions at slightly different time scales, this signal reasonably results from the formation of charge transfer (CT) excitons or free charge carriers photogenerated in (NDI-DAE)_2_PbI_4_ with the hole and electron in the inorganic and organic layers, respectively. While the formation of CT excitons has been claimed for similar systems previously, it should be noted that the GSB or PIA features of TA are not fully understood in these materials.^40^ Thus, unambiguously distinguishing between charge carriers or excitons is not possible, and more evidence is needed to show whether photon absorption by the organic can contribute to the free charge carrier formation in (NDI-DAE)_2_PbI_4_.

To investigate the formation of long-lived free charge carriers by visible-light absorption in the organic layers, we performed flash-photolysis time-resolved microwave conductivity measurements (fp-TRMC). This highly sensitive and electrode-free technique^41^ is a powerful tool to investigate free charge carrier dynamics in the direction parallel to the substrate upon photoexcitation. By measuring the transient photoconductivity of the thin film, a product of the free charge carrier generation quantum yield (φ) and the sum of the (electron and hole) mobilities, μ, as the fraction sum mobility, φ∑μ, can be estimated (see the Supporting Information for full experimental methods). The obtained photoconductivity transients and calculated sum mobilities for different photon fluxes are displayed in Figure 2d for (NDI-DAE)_2_PbI_4_ at excitation wavelengths of 420 and 530 nm. For the 420 nm case, a photoexcited φ∑μ of 3 × 10^–4^ cm^2^ V^–1^ s^–1^ at 3 × 10^13^ absorbed photon flux per cm^2^ is estimated. The measured sum mobility follows the characteristic dependency with light intensity: as the absorbed photon flux increases, increased exciton–exciton interactions cause a reduction in φ∑μ (Figure 2d, inset). We note that the time scale for the transfer of photogenerated electrons in the perovskite layer to the organic layer is a few picoseconds, as suggested by the TA dynamics discussed above, which is beyond the few nanosecond time-resolution of the fp-TRMC measurement. Effectively, this means that photoexcited electrons will already be localized on the organic at the onset of the fp-TRMC measurement. Given the generally lower charge carrier mobility in organic semiconductors compared to inorganics, this electron localization is expected to result in lower measured carrier mobility compared to that observed in conventional (BA)_2_PbI_4_ thin films, where photogenerated electrons remain in the CB of the inorganic slabs and mobilities of more than 1 order of magnitude higher are observed (*vide infra*).

The photoconductivity transient of the (NDI-DAE)_2_PbI_4_ at an excitation wavelength of 420 nm (Figure 2d, main panel) fits to a biexponential with short (τ_1_ = 18 ns) and long (τ_2_ = 484 ns) decay constants. This stands in contrast to (BA)_2_PbI_4_, where transient decays with a single time constant of around 17 ns were observed and ascribed to exciton relaxation in the inorganic layer.^42^ The formation of free charges across the organic–inorganic interface in the (NDI-DAE)_2_PbI_4_ reasonably causes the prolonged photoconductivity and suggests that an additional free charge carrier relaxation mechanism occurs in the (NDI-DAE)_2_PbI_4_ thin film.

Notably, comparable mobilities (4 × 10^–4^ cm^2^ V^–1^ s^–1^ at 10^13^ absorbed photons per cm^2^) were obtained when (NDI-DAE)_2_PbI_4_ was photoexcited at a wavelength of 540 nm, which is predominantly absorbed by the organic. This accords with the TA results, further suggesting that photon absorption by the NDI-DAE can contribute to the light harvesting process and free charge carrier generation.

**Figure 3:**
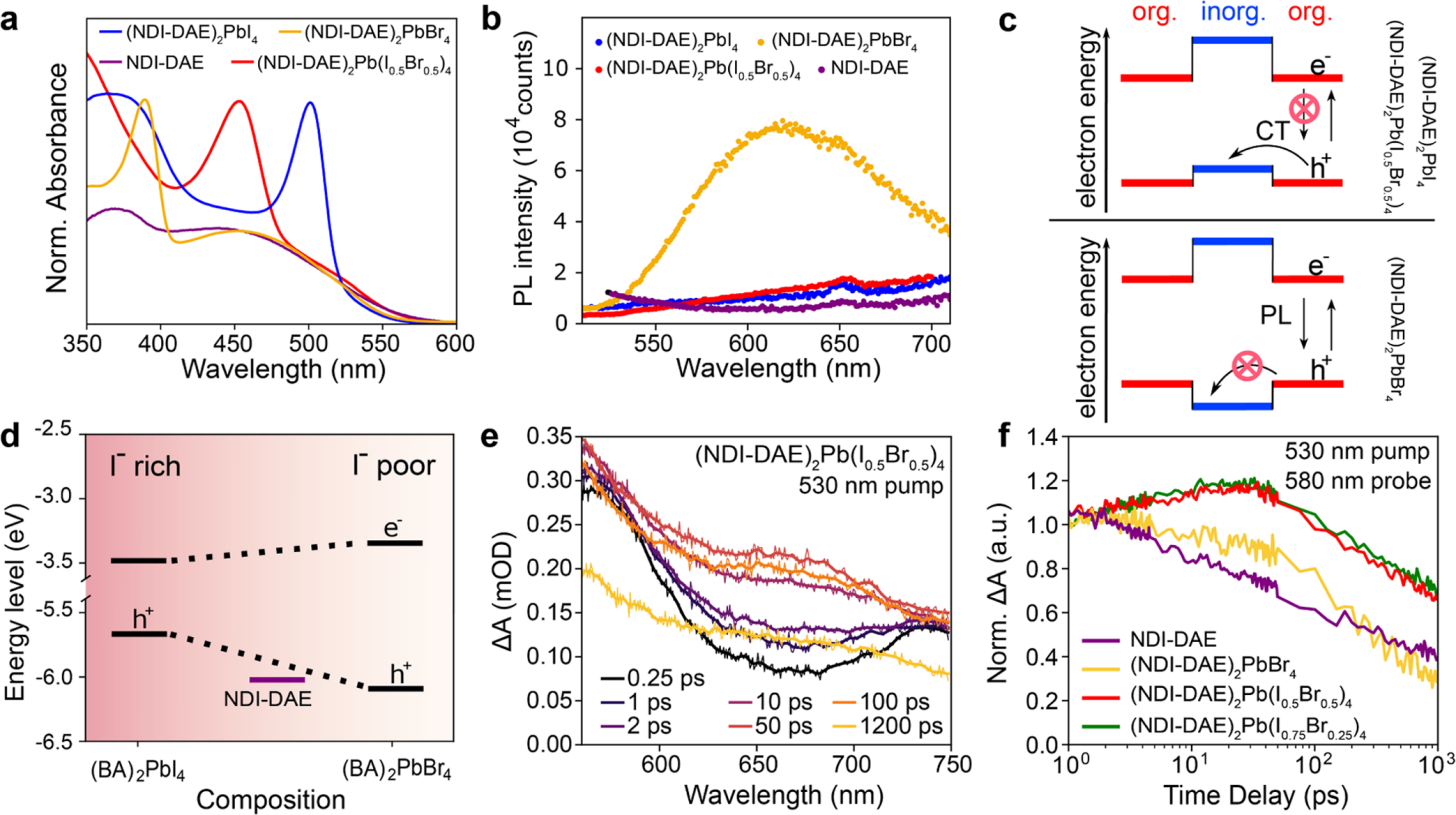
(a) UV–visible-light absorbance of thin films of NDI-DAE LHOIPs with different halide compositions as well as the UV–visible-light absorbance of the NDI-DAE salt. (b) Photoluminescence (PL) emission of the thin films from (a) excited at 480 nm. (c) Schematic energy levels of the (NDI-DAE)_2_PbI_4_ and 1:1 mixed I:Br version (top) show the type-II heterostructure arrangement where the PL is quenched due to hole transfer from the organic chromophore to the inorganic perovskite layer. While the inverse type I structure of the (NDI-DAE)_2_PbBr_4_ (bottom) does not allow this hole transfer. (d) VB energies were obtained from UPS spectroscopy for BA_2_PbX_4_ reference materials with respect to the HOMO of NDI-DAE. (e) TA spectra of (NDI-DAE)_2_Pb(I_0.5_Br_0.5_)_4_ after photoexcitation at 530 nm and the resulting rise of the PIA band. (f) Dynamics of the rise of the PIA at 580 nm in (NDI-DAE)_2_Pb(I_*x*_Br_1–*x*_)_4_ with *x* = 0, 0.5, 0.75 compared to the NDI-DAE salt.

To better support the role of light harvesting by the organic in our LHOIP structure and further demonstrate the optoelectronic versatility of NDI-DAE, different halide compositions of the inorganic layer were explored. Indeed, varying the halide in the inorganic layer effectively modifies the band gap energy of the inorganic layer while the electronic structure of the organic layer remains unchanged.^43^ By changing the halide to Br^–^, thin films of (NDI-DAE)_2_PbBr_4_ showed a significant blue shift in the excitonic absorption, as illustrated in Figure 3a. Consequently, only the NDI-DAE organic layer absorbs in the wavelength range between 420 and 560 nm, as supported by control films of (BA)_2_PbBr_4_, which showed negligible photon absorption in this range (Figure S7). Further, using a mixed halide composition (NDI-DAE)_2_Pb(I_0.5_Br_0.5_)_4_ gave a single intermediate excitonic absorption peak, as shown in Figure 3a. This tunability in the excitonic absorption peak was found to be similar in films of (BA)_2_Pb(I_*x*_Br_1–*x*_)_4_, as shown in Figure S7. It is noted that the interplane distances were not substantially changed for all investigated halide mixtures, as indicated by the XRD (Figure S8 for BA and Figure S9 for NDI-DAE).

To gain more insight into the effect of the halide composition on the optoelectronic properties of the NDI-DAE-based materials, PL spectra of thin films of the pure bromide-based ((NDI-DAE)_2_PbBr_4_) LHOIP, the pure iodide based ((NDI-DAE)_2_PbI_4_) LHOIP, and the LHOIP containing a 1:1 halide mix ((NDI-DAE)_2_Pb(I_0.5_Br_0.5_)_4_), in the range of 550–700 nm, were acquired (excitation wavelength of 400 nm, Figure 3b). The (NDI-DAE)_2_PbBr_4_ film shows a strong and broad emission band, with a maximum at 600 nm. By selectively exciting the organic layer in this film at 480 nm, where minimal absorption of the inorganic is expected, the emission band at 600 nm remains the same (Figure S10), indicating that the emission arises from the organic layer. Indeed, the PL emission corresponds well to a similar naphthalene-iminoimide dye as previously reported in the solution.^35^ However, we note that a thin film of pure NDI-DAE salt resulted in no observable PL emission, likely due to aggregation-induced quenching when the organic component is not incorporated into a layered perovskite structure (or in a dilute solution), as supported by PL measurements acquired with NDI-DAE at various concentrations (see Figure S11). This strongly suggests that (NDI-DAE)_2_PbBr_4_ forms an inverted (or reversed) type I nanoheterostructure where hole transfer from the organic semiconductor layer to the inorganic perovskite slab is not favored, facilitating radiative recombination in the organic layer. Interestingly, photon absorption by the inorganic slabs, followed by resonance energy transfer to the organic material, could also lead to the observed PL emission in this case.

In contrast, no PL emission in the range of 550–700 nm is observed for the (NDI-DAE)_2_Pb(I_0.5_Br_0.5_)_4_ and (NDI-DAE)_2_PbI_4_ thin films when excited at 400 nm. This is expected given the energy level alignment of the organic and inorganic layers, wherein after excitation of the inorganic, hole injection to the organic layer and spatial separation of the charge carriers are favored, as shown schematically in Figure 3c. The proposed electronic nanoheterojunction configurations of these materials are supported by ultraviolet photoelectron spectroscopy (Figures S12 and S13), with which the respective CB energies of (BA)_2_PbI_4_ and (BA)_2_PbBr_4_ are estimated and are found to be consistent with previous reports.^44^ Schematic comparison of the HOMO of the NDI-DAE and the CB, VB levels of the (BA)_2_Pb(I_*x*_Br_1–*x*_)_4_ system as shown in Figure 3d, illustrates that the VB value changes more strongly with the halide composition of the inorganic layer and confirms that hole transfer from the HOMO of the NDI-DAE to the inorganic should not be possible for iodide-poor (Br-rich) inorganic perovskite slabs.

TA spectroscopy brings further insight into the charge transfer dynamics in the mixed halide LHOIP systems. Similar to the case of (NDI-DAE)_2_PbI_4_, a broad PIA in the range between 580 and 700 nm was observed in (NDI-DAE)_2_Pb(I_0.5_Br_0.5_)_4_ excited at 530 nm, as displayed in Figure 3e. The dynamics of the PIA rise at 580 nm is shown in Figure 3f. The rise of the PIA, which can be accordingly attributed to the absorption of a CT state or free charge carriers in the (NDI-DAE)_2_Pb(I_0.5_Br_0.5_)_4_, is on the same time scale as observed for the (NDI-DAE)_2_PbI_4_ and an LHOIP with another intermediary halide composition, namely, (NDI-DAE)_2_Pb(I_0.75_Br_0.25_)_4_. Moreover, TA spectra of the (NDI-DAE)_2_Pb(I_0.5_Br_0.5_)_4_ after excitation at 525 nm (Figure S14) show again a characteristic GSB at around 420–460 nm, suggesting hole injection from the organic to the inorganic layer, which was not observed in the (NDI-DAE)_2_PbBr_4_ films. Likewise, the presence of a PIA in the 580–700 nm range was not observed upon the photoexcitation of (NDI-DAE)_2_PbBr_4_ films (Figure S15).

**Figure 4:**
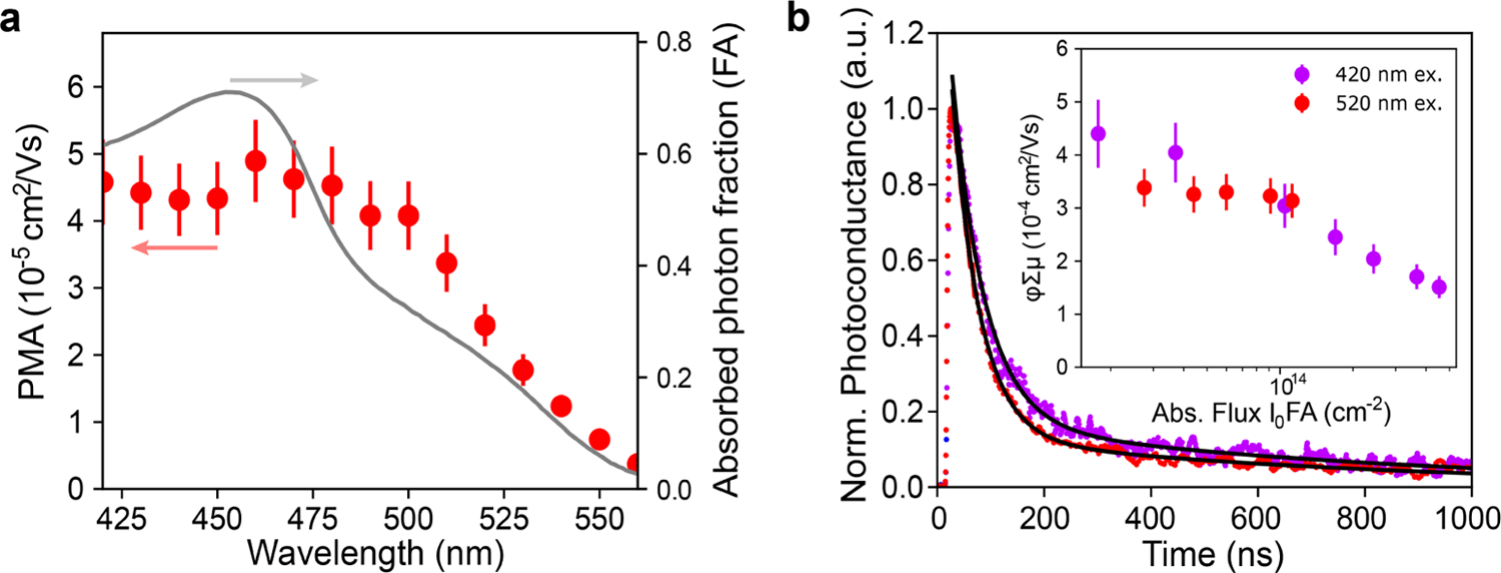
(a) Photoexcited mobility action spectrum of (NDI-DAE)_2_Pb(I_0.5_Br_0.5_)_4_. Peak photoconductivity (red closed circles, left axis) is normalized by incident light intensity but not absorptance (gray solid line, right axis), showing the shape of the absorbance indicating an electronic connection between the perovskite and the organic. The error bars are calculated based on uncertainties given by the photon fluence. (b) fp-TRMC transient and peak photoconductivity of (NDI-DAE)_2_Pb(I_0.5_Br_0.5_)_4_ at 420 nm (purple) and 520 nm (red) excitation wavelengths.

The above results confirm the tunability of the nanoheterojunction type by changing the halide composition in our (NDI-DAE)_2_Pb(I_*x*_Br_1–*x*_)_4_ films, giving further opportunity to strengthen evidence of the role of light absorption on free charge carrier formation in these materials. Indeed, while the TA and fp-TRMC on (NDI-DAE)_2_PbI_4_ strongly suggest photoexcitation of the organic leads to hole transfer from the organic to the inorganic and the formation of a CT state or free carriers, a slight photon absorption by the inorganic slab cannot be fully discounted in the range of 520–600 nm in (NDI-DAE)_2_PbI_4_. However, in the mixed halide material (NDI-DAE)_2_Pb(I_0.5_Br_0.5_)_4_, the organic layer can be unambiguously selectively excited. Using the fp-TRMC technique, (NDI-DAE)_2_Pb(I_0.5_Br_0.5_)_4_ films were excited at different wavelengths and the maximum value of the measured photoconductance was normalized by incident light intensity (not absorbance, see Experimental Methods in Supporting Information) to give a photoexcited mobility action (PMA) spectrum, which is proportional to the photogenerated free charge carrier concentration. Figure 4a compares the PMA with the fraction of photons absorbed (FA) in the mixed halide thin film, and a good correspondence is observed. Indeed, an explicit contribution to free charge carrier mobility from photons in the range from 500 to 560 nm is observed.

Furthermore, excitation of (NDI-DAE)_2_Pb(I_0.5_Br_0.5_)_4_ at 420 nm, where the photons are absorbed by both the inorganic perovskite layer and the organic, and at 520 nm, where photons are only absorbed by the organic layer gave the photoconductance transients and φ∑μ vs flux plots in Figure 4b. Here, excitation at 420 nm leads to a measured φ∑μ of 4 × 10^–4^ cm^2^ V^–1^ s^–1^ at 3 × 10^13^ absorbed photon flux, which is slightly higher compared to (NDI-DAE)_2_PbI_4_. Importantly, comparable φ∑μ was obtained when free charge carriers were generated at 520 nm. The photoconductance transients, when excited at 420 and 520 nm, strongly overlap, indicating similar decay rates and decay processes when only the organic layer or both organic and inorganic layers are excited. Overall, these findings suggest that any imperfections of the halide distribution in mixed halide layered perovskite, which have been previously reported to result in pseudobinary alloys, do not greatly affect the thin film optoelectronic properties. In contrast, we note that BA-based LHOIPs with mixed halides show reduced PL intensity and increased Stokes shift (Figure S16), likely due to electron and hole transfer from bromide-rich to the iodide-rich domains. This electron funneling effect strongly affects the determined photoconductivity of BA-based mixed halide films. While a decrease in determined photoconductivity in the (BA)_2_PbBr_4_ compared to the (BA)_2_PbI_4_ can be attributed to increased exciton binding energies and resulting lower free charge carrier generation yield (φ), the reduced photoconductivity in (BA)_2_Pb(I_*x*_Br_1–*x*_)_4_ (*x* = 0.25, 0.50, and 0.75, Figure S17) reasonably arises from reduced mobility due to the inhomogeneous energy landscape in the inorganic layer.^45−48^ This makes comparing the estimated mobilities of (BA)_2_Pb(I_*x*_Br_1–*x*_)_4_ to that of (NDI-DAE)_2_Pb(I_0.5_Br_0.5_)_4_ unsuitable for drawing conclusions regarding the locations of the free charge carriers. Therefore, we measured the free charge carrier generation in the reverse type I heterostructure (NDI-DAE)_2_PbBr_4_ film. The fp-TRMC data of this material (Figure S18) show a peak φ∑μ 3 times lower than those of (NDI-DAE)_2_Pb(I_0.5_Br_0.5_)_4_ and (NDI-DAE)_2_PbI_4_ upon excitation at 500 nm. Thus, it is reasonable to conclude that the generated free charge carriers excited at 500 nm in (NDI-DAE)_2_Pb(I_0.5_Br_0.5_)_4_ and (NDI-DAE)_2_PbI_4_ are in higher mobility inorganic slabs, but they arise solely from the photoexcitation of the organic layer. This is further supported by the free charge carrier decay dynamics of (NDI-DAE)_2_PbBr_4_, which show comparable free charge carrier decay properties as the as-spun coated NDI-DAE salt (Figure S19). It should be noted that halide segregation in the inorganic slabs of the mixed (NDI-DAE)_2_Pb(I_0.5_Br_0.5_)_4_ material is not expected, based on previous reports, which show this effect to be appreciable only for quasi-layered (*n* > 1) materials under long-term, high light intensity conditions.^45,49^ Accordingly, the observed low mobility of (NDI-DAE)_2_PbBr_4_ when only the chromophore is excited represents the mobility of the carriers through the organic layer, which is suggested to be a limiting factor in charge transport in type II heterostructures based on the layered perovskites. Indeed, while the type II heterostructure LHOIP, like the (NDI-DAE)_2_PbI_4_ examined herein, has the advantage of charge separation between the organic and inorganic layer, increasing the charge transport in the organic layer remains a challenge for future development of these materials toward high-performance device applications.

## Conclusions

Engineering the molecular structure of naphthalenediimide-based cations for layered “2D” perovskites afforded a new lead-halide-based Ruddlesden–Popper material incorporating a visible-light-absorbing organic. Thin films of (NDI-DAE)_2_PbI_4_ showed significant photoluminescence quenching and transient absorption dynamics consistent with the production of a charge transfer state or free charge carriers at excitation wavelengths predominately absorbed by either the inorganic or organic layers. Estimation of the electronic energy levels together with time-resolved microwave conductivity further support the formation of a type II nanoheterostructure where photoexcitation yields free electrons and holes in the organic and inorganic layers, respectively, with sum mobilities up to 4 × 10^–4^ cm^2^ V^–1^ s^–1^. Tuning the halide composition modifies the band gap of the inorganic layer, converting the nanoheterostructure to inverted (reversed) type I with high bromide content (as shown by UPS spectroscopy and photoluminescence) where photon absorbance by the inorganic results in energy transfer to the organic followed by radiative recombination. For intermediate Br, I concentrations in (NDI-DAE)_2_Pb(I_*x*_Br_1–*x*_)_4_ films, the selective photoexcitation of the organic is demonstrated, and transient absorption spectra together with microwave conductivity measurements provide strong evidence that visible-light absorption by the NDI-DAE cation generates separated free carriers via hole transfer to the inorganic layer. While the relatively low electron mobility in the organic layer offers a challenge for further development of this class of materials, the potential to tune the harvesting of visible photons and transport complementary charges in layers of a nanoheterojunction offers a promising outlook to this class of hybrid semiconductors for future photovoltaic or photodetector applications.

## Supporting Information Available

Full Experimental Methods, Figures S1–S16. (PDF)
