# Supplementary material for: Free Charge Carrier Generation by Visible-Light-Absorbing Organic Spacers in Ruddlesden–Popper Layered Perovskites
Source: J Am Chem Soc. 2024 Sep 24;146(40):27770–8. doi: 10.1021/jacs.4c09706 (PMC11467899; doi:10.1021/jacs.4c09706)
Supplement: Supplementary file 1 — ja4c09706_si_001.pdf [file ja4c09706_si_001.pdf]

Supporting information for

Free charge carrier generation by visible light absorbing organic spacers in Ruddlesden-Popper layered perovskites

Simon Nussbaum[a], Demetra Tsokkou[b], Aaron T. Frei[c], Dennis Friedrich[d], Jacques-E. Moser[c], Natalie Banerji[b], Jun-Ho Yum[a], Kevin Sivula*[a]*

[a] Laboratory for Molecular Engineering of Optoelectronic Nanomaterials, Institute of Chemical Sciences and Engineering (ISIC), École Polytechnique Fédérale de Lausanne (EPFL), 1015 Lausanne, Switzerland

[b] FemtoMat Research Group, Department für Chemie, Biochemie und Pharmazie, University of Bern, Freiestrasse 3, 3012 Bern, Switzerland

[c] Photochemical Dynamics Group, Institute of Chemical Sciences and Engineering (ISIC), École Polytechnique Fédérale de Lausanne (EPFL), 1015 Lausanne, Switzerland

[d] Institute for Solar Fuels, Helmholtz Zentrum Berlin für Materialien und Energie, Hahn-Meitner-Platz 1, 140109 Berlin, Germany

E-mail: Junho.yum@epfl.ch, Kevin.sivula@epfl.ch

Experimental Methods

Chemicals

N-Boc-1,4-butanediamine (95\%, CAS: 68076-36-8) and Naphthalene-1,4,5,8-tetracarboxylic dianhydride (95\%, CAS: 81-30-1) was purchased from ABCR. Perovskite precursor solutions were prepared with lead (II) iodide (99.99\%, CAS: 10101-63-0) and lead(II) bromide (>98 \%, CAS: 10031-22-8) from TCI.

Synthesis of the NDI-DAE cation

NDI-DAE-Boc

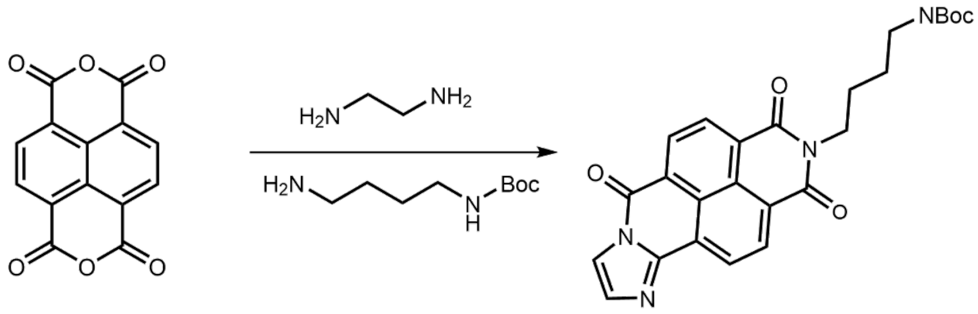

Scheme S1. Synthetic route to BOC-protected NDI-DAE spacer.

5 g (18.7 mmol) of Naphthalenetetracarboxylic dianhydride were dissolved in 80 ml of dry DMF under an argon atmosphere. Then 1.5 ml (1.08 g, 18.74 mmol, 1 eqv.) ethylenediamine and 3.53 g (18.76 mmol, 1 eqv.) of N-Boc-1,4-diaminobutane. were added. The reaction mixture was stirred for 24 h at 120 °C. The crude product was precipitated by adding 100 ml water to the reaction and obtained after subsequent filtration of the precipitant. Additionally, the precipitant was redissolved in DCM and washed with H_2O (5x 50 ml) to remove remaining DMF. The desired product was obtained by silica gel column purification using dichloroethyl (DCM):methanol (99.5:0.5) as eluent. Yield: 2.031 g (2.24 mmol, 12 %).

^{1}H NMR (400 MHz, $CDCl_3$-d) δ 8.89 (d, J = 7.6 Hz, 1H), 8.81 – 8.72 (m, 4H), 7.94 (d, J = 1.6 Hz, 1H), 7.45 (d, J = 1.6 Hz, 1H), 4.63 (br, 1H), 4.26 – 4.18 (m, 2H), 3.21 (q, J = 6.5 Hz, 2H), 1.86 – 1.74 (m, 2H), 1.66 (m, 2H), 1.43 (s, 9H).

^{13}C NMR (101 MHz, $CDCl_3$-d) δ 163.05, 162.91, 158.34, 156.09, 144.58, 133.20, 132.22, 130.74, 128.37, 127.61, 126.68, 125.78, 125.47, 124.79, 123.79, 116.49, 66.31, 53.57, 40.52, 28.56, 27.78, 25.58.

HRMS (ESI/QTOF) m/z: [M + Na]+ Calcd for $C_{25}H_{24}N_4NaO_5^+$ 483.1639; Found 483.1638

EA: Anal. Calcd for $C_{25}H_{24}N_4O_5$: C, 65.21; H, 5.25; N, 12.17. Found: C, 64.93; H, 5.21; N, 12.09.

NDI-DAE-salt

After evaporation of the solvent, the solids were redissolved in Methanol and HI (57%) was added while cooling the reaction mixture in an ice bath. After 1 h, the solid ammonium iodine salt precipitated out. Further material was precipitated by addition of diethylether.

^{1}H NMR (DMSO-d6, 400 MHz): δ 1.64 (7H, d, J = 5.7 Hz), 1.73 (7H, m), 2.51 (13H, quint, J = 1.9 Hz), 2.87 (8H, d, J = 7.2 Hz), 3.36 (27H, s), 4.04 (9H, t, J = 6.7 Hz), 7.43 (4H, d, J = 1.5 Hz), 7.64 (7H, m), 8.00 (4H, d, J = 1.5 Hz), 8.36 (1H, s), 8.38 (3H, s), 8.42 (3H, s), 8.44 (1H, s), 8.51 (4H, d, J = 7.6 Hz), 8.61 (4H, d, J = 7.6 Hz)

^{13}C NMR (101 MHz, DMSO-d6) δ 162.41, 162.28, 157.69, 143.70, 132.72, 131.47, 131.21, 130.13, 127.08, 126.36, 126.14, 124.87, 124.39, 123.60, 122.68, 116.64, 38.71, 24.76, 24.63.

Thin film formation

(NDI-DAE)$_2$PbI$_4$ layered perovskite films were synthesized by spin-coating a 0.4 M NDI-DAE-I + 0.2M PbI$_2$ in DMSO solution on SiO$_2$ (quartz) substrates at 2000 rpm for 30 seconds. The perovskite crystalline thin films were formed upon thermal annealing at 200 °C for 10 minutes.

Basic characterization methods

Thin film X-ray diffraction (XRD) measurements were taken in Bragg-Brentano geometry using non-monochromatic Cu-Kα1 radiation on a Bruker D8 Vario instrument equipped with a LynxEYE XE detector. Grazing-incidence wide-angle X-ray scattering experiments were carried out at the European Synchrotron Radiation Facility (in Grenoble, France) at beamline BM01. The samples were measured under ambient conditions with a 0.6506 Å (19.06 keV) X-ray source and a beam size of 0.5×0.5 mm^2 onto the sample coming in at an incidence angle of α = 0.25°. UV-Visible light absorption measurements were carried out with a UV-3600 Shimadzu spectrometer.

Transient absorption spectroscopy

Femtosecond transient absorption measurements were performed using a Ti:Sapphire amplifier system (Astrella, Coherent) with output pulses of 35 fs duration, 800 nm wavelengths and 1 kHz repetition rate. For the TA measurements the beam is split intto two components, the pump and the probe. To convert the wavelength of the pump beam to 530 nm an optical parametric amplifier (Opera, Coherent) is used and a BBO crystal was used to generate 400 nm by doubling the frequency of the fundamental beam. The broad spectrum of the probe beam is achieved by strongly focusing the fundamental light into a sapphire crystal to generate white light. The optical path of the probe pulses compared to the one of the pump pulses is adjusted with a computer control translation stage. Probe beam is splited into a reference beam and a signal beam. Pump and signal probe beams are spatially and temporally overlapped on the sample position, where the size of the pump beam was 3-4 times bigger the probe beam size to ensure that we probe uniform photoexcited species distribution. The transmitted probe pulses are directed and spectrally dispersed in a home-built prism spectrometer (Entwicklungsbüro Stresing, Berlin) and detected with back-thinned Silicon CCD camera (Hamamatsu S07030-0906) to probe the visible wavelengths from 480 nm to 750 nm. The pump pulses were chopped at 500 Hz to measure of transmission change of the probe pulses and increase the signal to noise ratio. The measurements were performed for a probe pulses polarization at magic angle with respect to the one of the pump pulses to avoid any polarization effects.

Flash-photolysis time-resolved microwave conductivity measurements

TRMC measurements were performed by mounting the samples in a microwave cavity cell and placed in a setup similar to the one described elsewhere. A voltage-controlled oscillator (SiversIMA VO3262X) generated the microwaves (X-band region, 8.4-8.7 GHz). During the measurements, a change in the microwave power ($\frac{\Delta P}{P}$) reflected by the cavity upon sample excitation by 3 ns (full-width at half-maximum) pulses of a wavelength tunable optical parametric oscillator (OPO) coupled to a diode-pumped Q-switched Nd:YAG laser at a

wavelength of 420 nm (50 Hz repetition rate) was monitored and correlated to the photoinduced change in the conductance of the sample, ΔG. The free charge carriers cause the change of the microwave power probe allowing assessment of the sum of their mobilities, μ, and the free charge-carrier generation quantum yield (φ) by the formula:

$$\phi \sum \mu = \frac{\Delta G}{\beta e I_0 F A}$$

where ΔG is proportional to the fractional change in reflected microwave power (ΔP/P), β is the ratio of the inner microwave cavity dimensions, and I_0FA is the number of absorbed photons (the incident light intensity, I_0 and the fraction of photons absorbed as the excitation wavelength, FA). As the microwave absorbance is highly sensitive only to free charge carriers, selectively exciting the organic allows us to elucidate whether the type II heterostructure can separate the CT excitons at the organic-inorganic interface.

To allow direct comparably between different samples in Figure 2d, we aimed to keep the absorbed photon flux constant by attenuating the incident laser beam with ND-filters. At wavelengths of high absorbance, more attenuation was required resulting in larger absolute errors on the incident photon flux and therefore larger uncertainty on the absorbed photon flux.

To generate the wavelength-dependent data for Figure 4a, the fraction sum mobility normalized by incident light intensity (not absorbance) using the following relation to give the photoexcited mobility action (PMA) spectrum.

$$\mathrm{PMA} = \frac{\Delta G}{\beta e I_0}$$

Supporting Figures

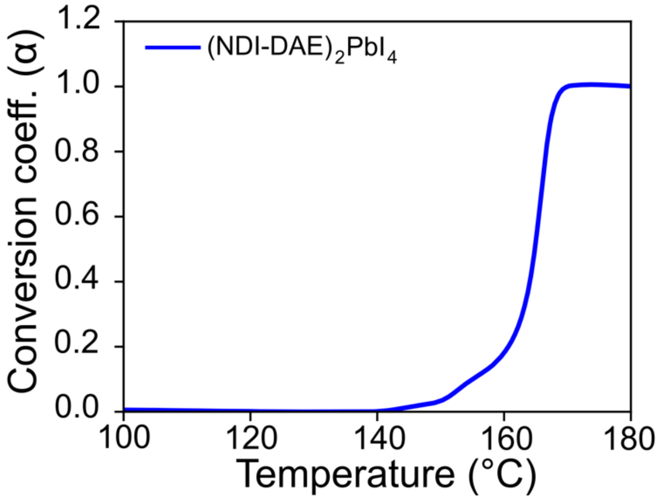

Figure S1. Evolution of the excitonic absorption band at the absorbance maxima while gradually increasing the temperature with a rate of 0.25 °C·s^{-1}, by using in-situ temperature dependent UV-vis spectroscopy (the experimental method is detailed in the ref[1]).

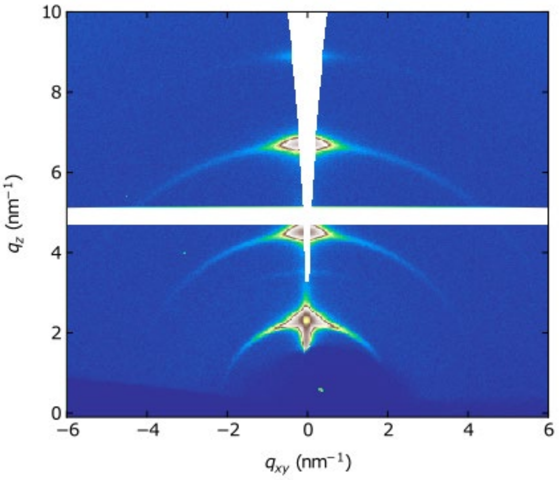

Figure S2. GIWAXS of (NDI-DAE)$_2$PbI$_4$ thin film measured at incident angle α = 0.25° with a 0.6506 Å (19.06 keV) X-ray source.

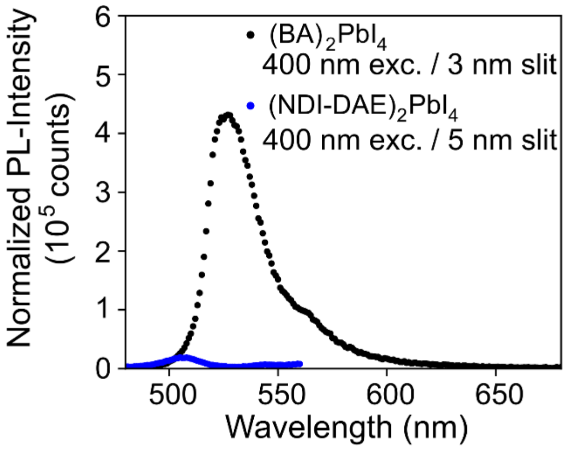

Figure S3. PL Intensity of thin films of $(NDI\text{-}DAE)_2PbI_4$ and $(BA)_2PbI_4$ (normalized to the absorbance at 400 nm). In order to obtain a strong signal for $(NDI\text{-}DAE)_2PbI_4$, the slit has to be increased. Films of similar thickness were used.

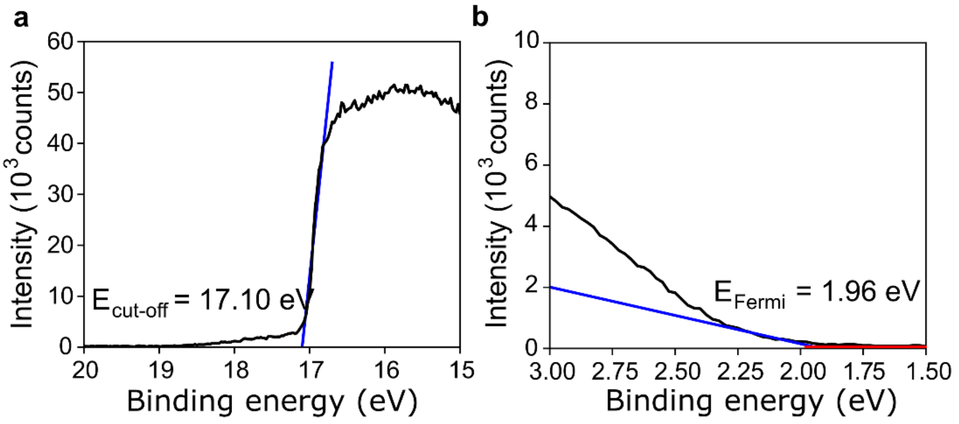

Figure S4. UPS Spectra of NDI-DAE.

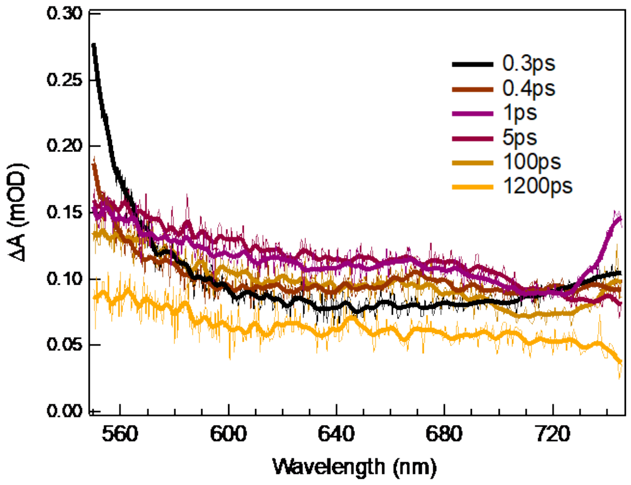

Figure S5. Transient absorption spectra of $(NDI\text{-}DAE)_2PbI_4$ films after excitation at 400 nm.

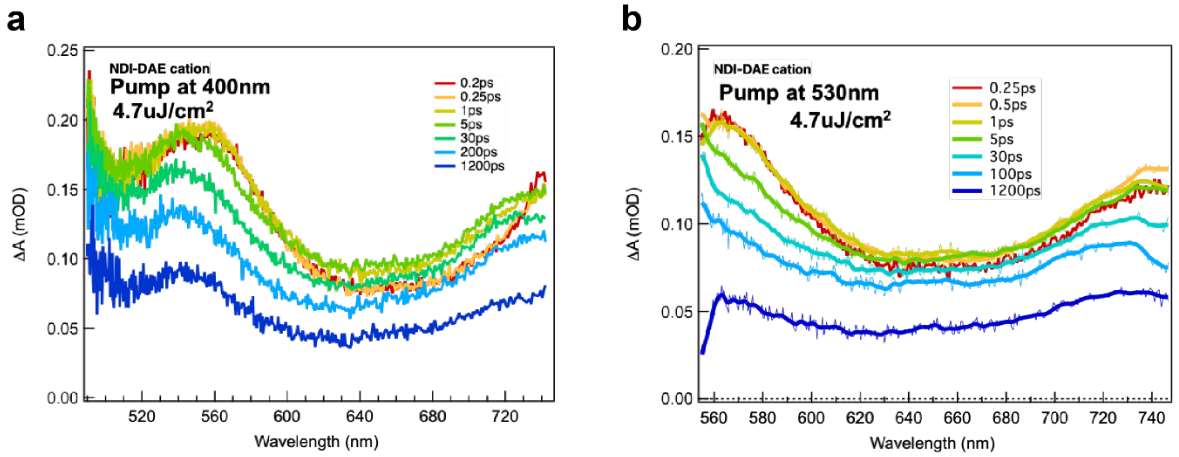

Figure S6. TA of thin films of pure NDI-DAE iodide salt excited at 400 nm and 530 nm.

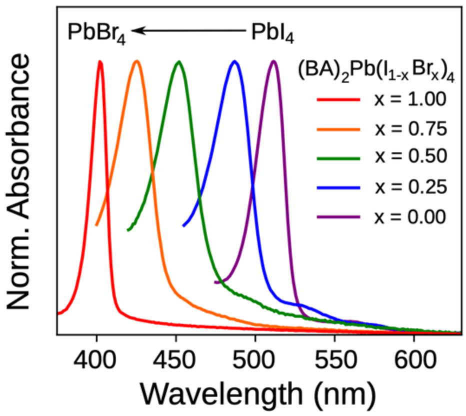

Figure S7. UV-visible absorption spectra of various $(BA)_2Pb(I_xBr_{1-x})_4$ films. For the sake of clarity, the spectra were normalized to the excitonic band maxima.

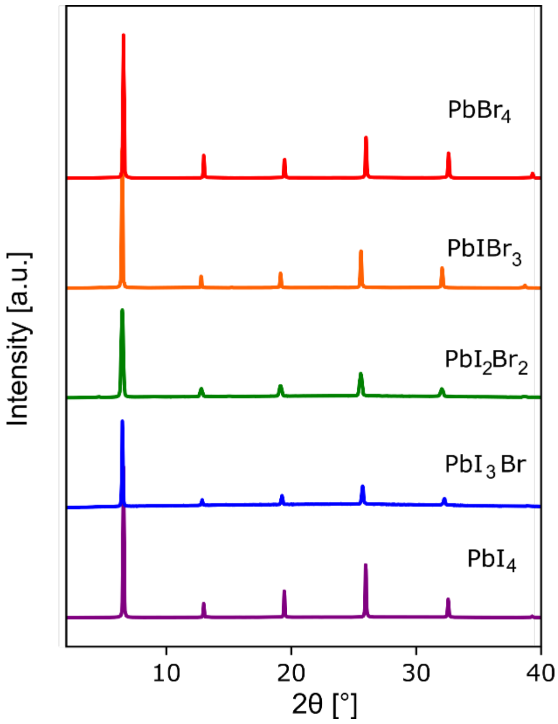

Figure S8. XRD patterns of investigated $(BA)_2Pb(I_xBr_{1-x})_4$ thin films prepared on SiO_2 substrates using a Cu $K_{\alpha 1}$ X-ray source.

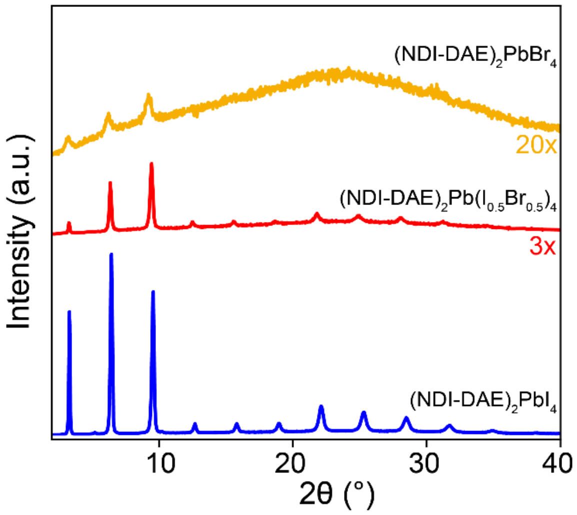

Figure S9. XRD if NDI-DAE-I, NDI-DAE-mix and NDI-DAE-Br using a Cu $K_{\alpha 1}$ X-ray source.

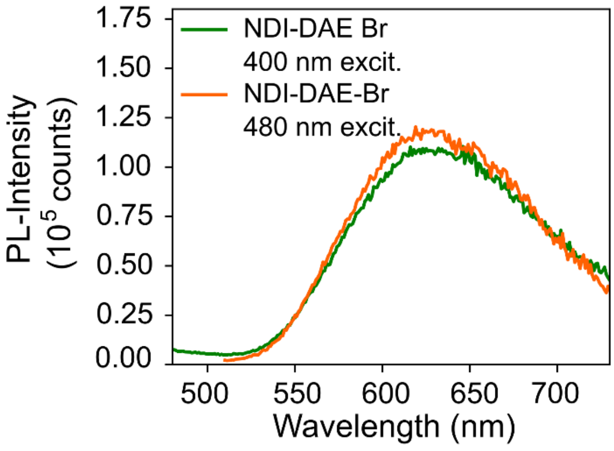

Figure S10. PL spectra of $(NDI\text{-}DAE)_2PbBr_4$.

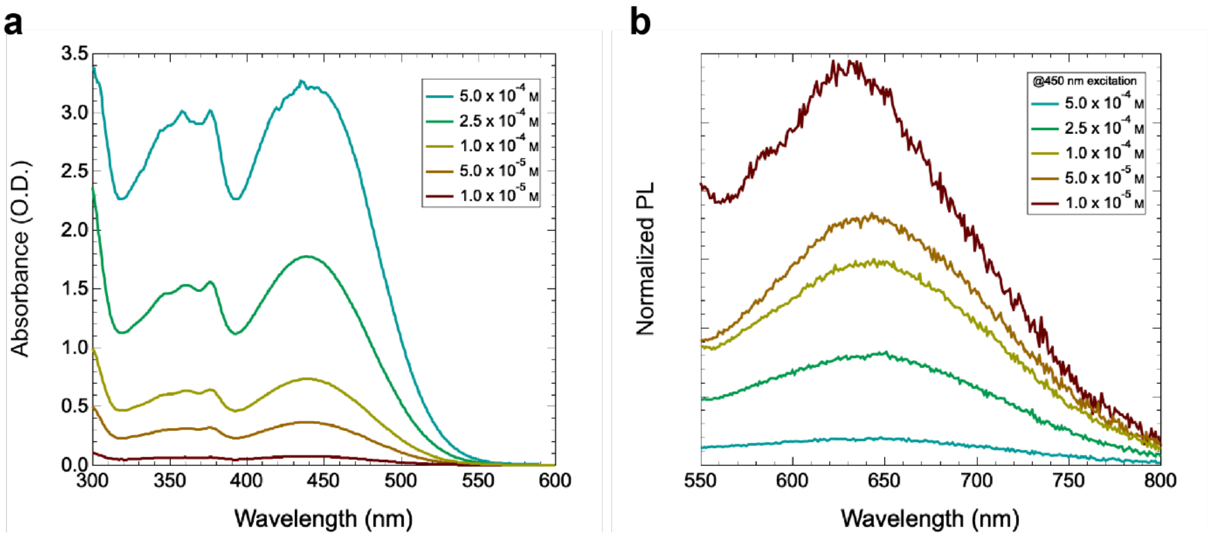

Figure S11. (a) UV-Vis absorbance and (b) PL (450 nm excitation, normalized to absorptance) of NDI-DAE solution in DMSO with different concentrations illustrating the decrease in PL at higher concentrations.

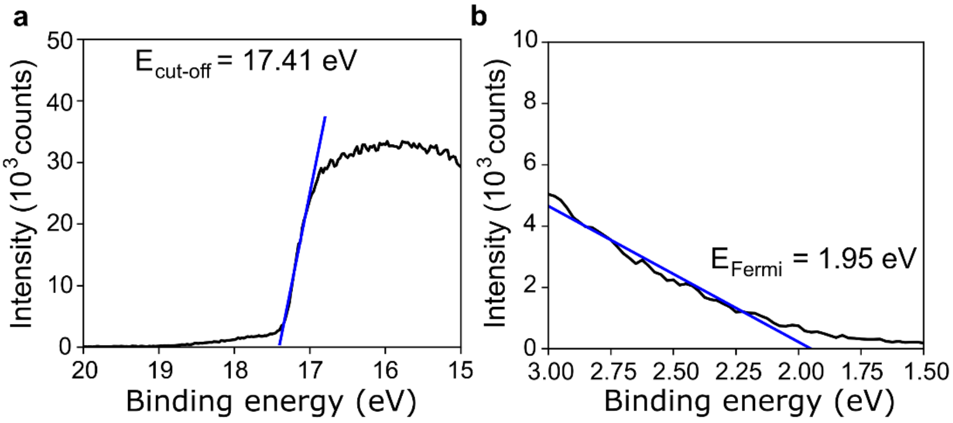

Figure S12. UPS of $(BA)_2PbI_4$.

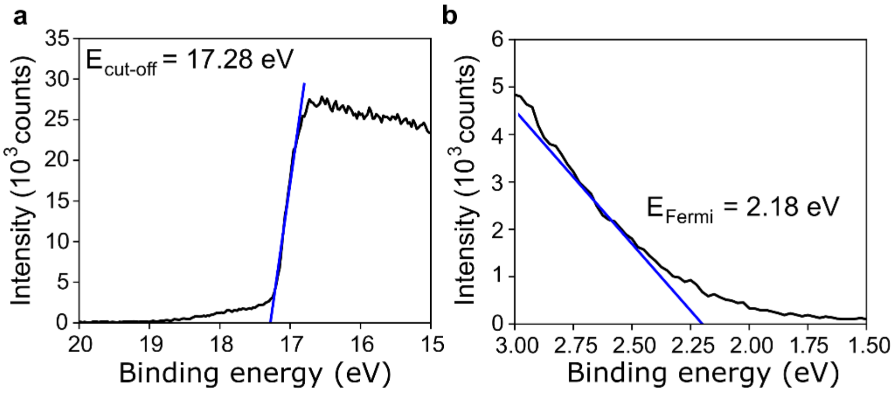

Figure S13. UPS of $(BA)_2PbBr_4$.

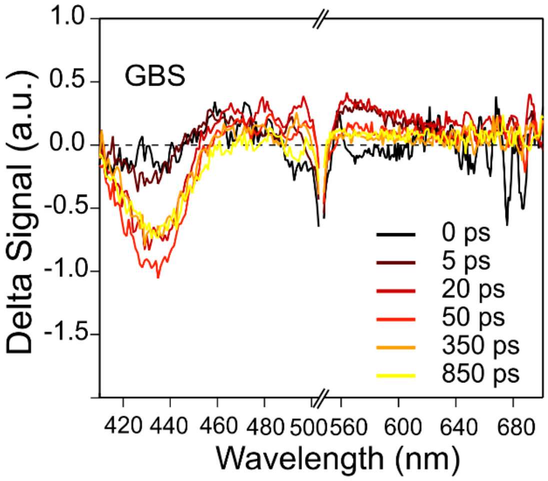

Figure S14. Transient absorption of $(NDI\text{-}DAE)_2Pb(I_{0.5}Br_{0.5})_4$ after photoexcitation at 525 nm.

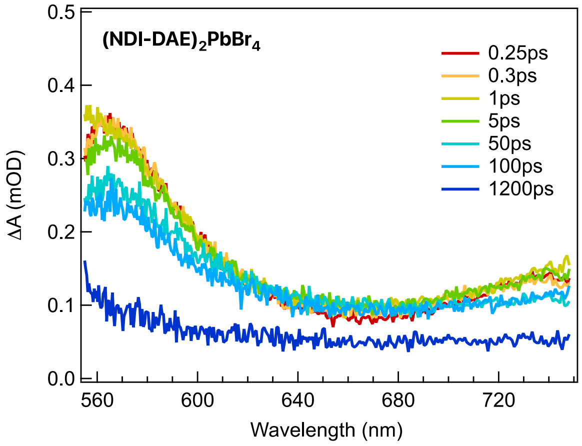

Figure S15. TA of $(NDI\text{-}DAE)_2PbBr_4$ cation excited at 530 nm.

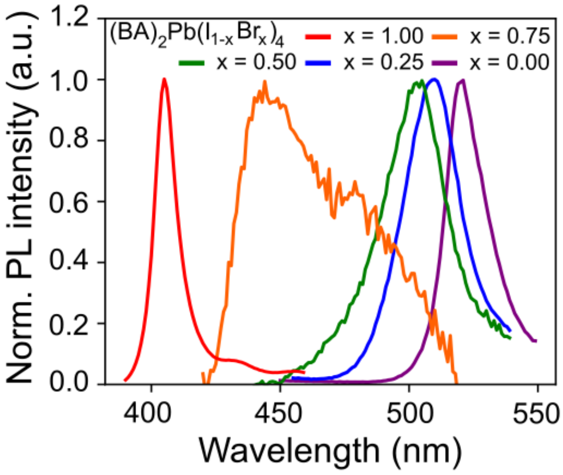

Figure S16. PL of investigated $(BA)_2Pb(I_xBr_{1-x})_4$ films excited at 400 nm.

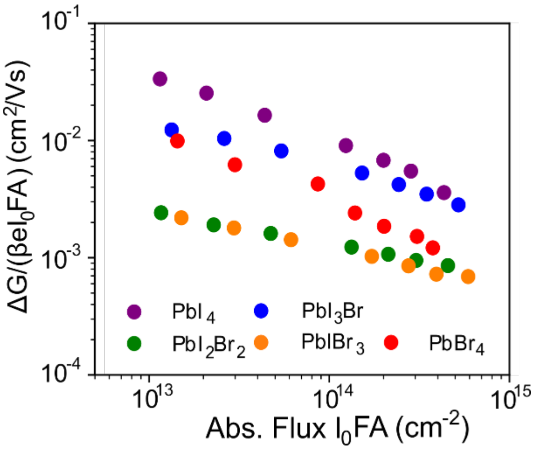

Figure S17. Mobility of $(BA)_2Pb(I_xBr_{1-x})_4$.

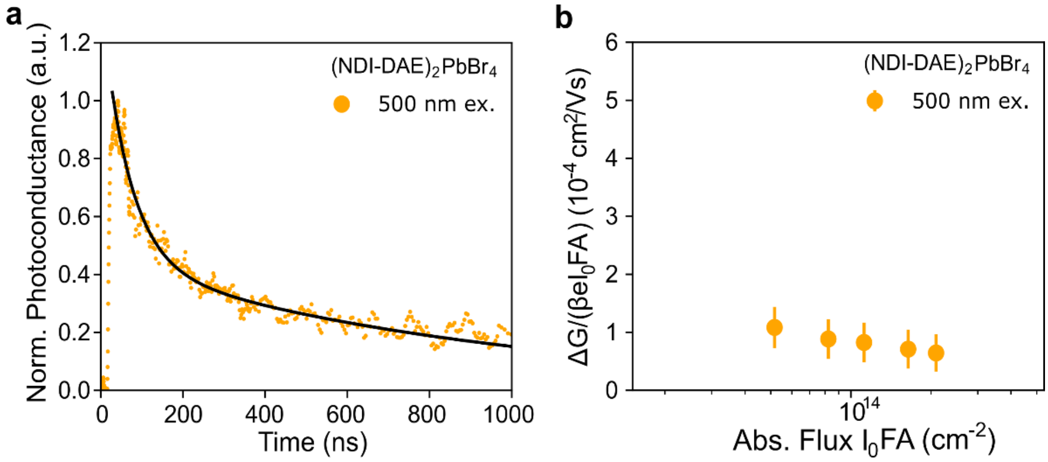

Figure S18. a) Transient and b) mobility of $(NDI\text{-}DAE)_2PbBr_4$ film when excited at 500 nm.

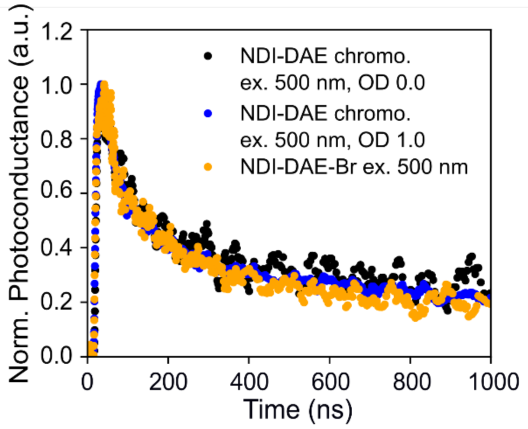

Figure S19. Normalized Photoconductance of NDI-DAE as spun-coated chromophore and $(NDI\text{-}DAE)_2PbBr_4$ (labeled as NDI-DAE-Br) excited at 500 nm.

NMR Spectra

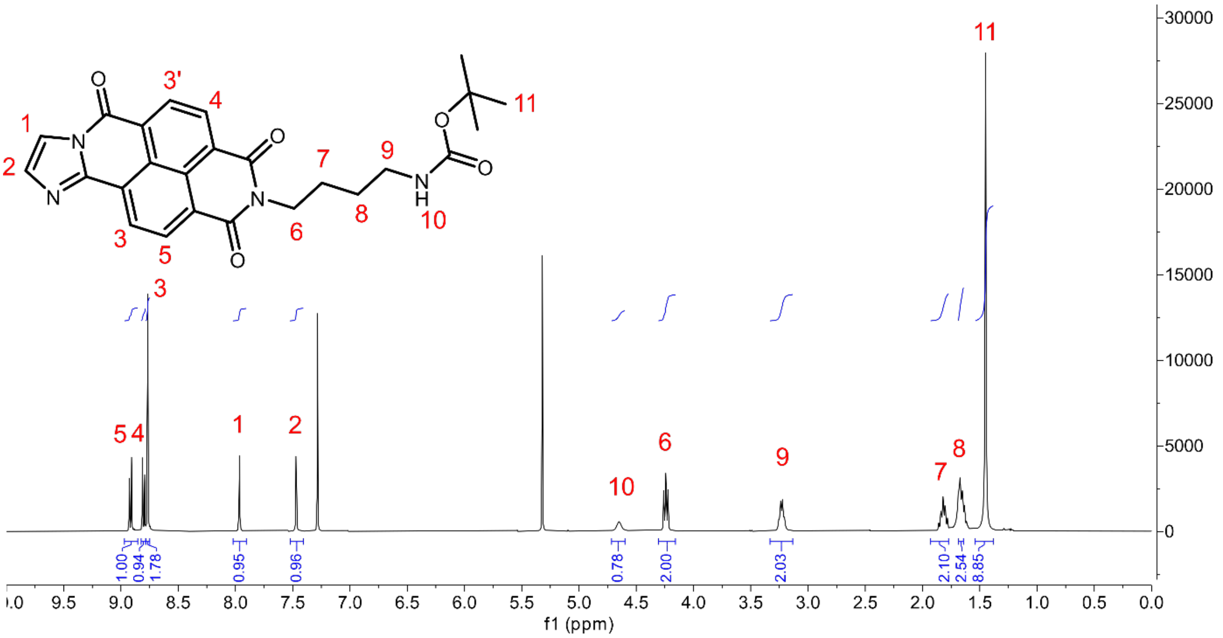

NMR 1: ^{1}H-NMR of NDI-DAE-Boc in $CDCl_3$.

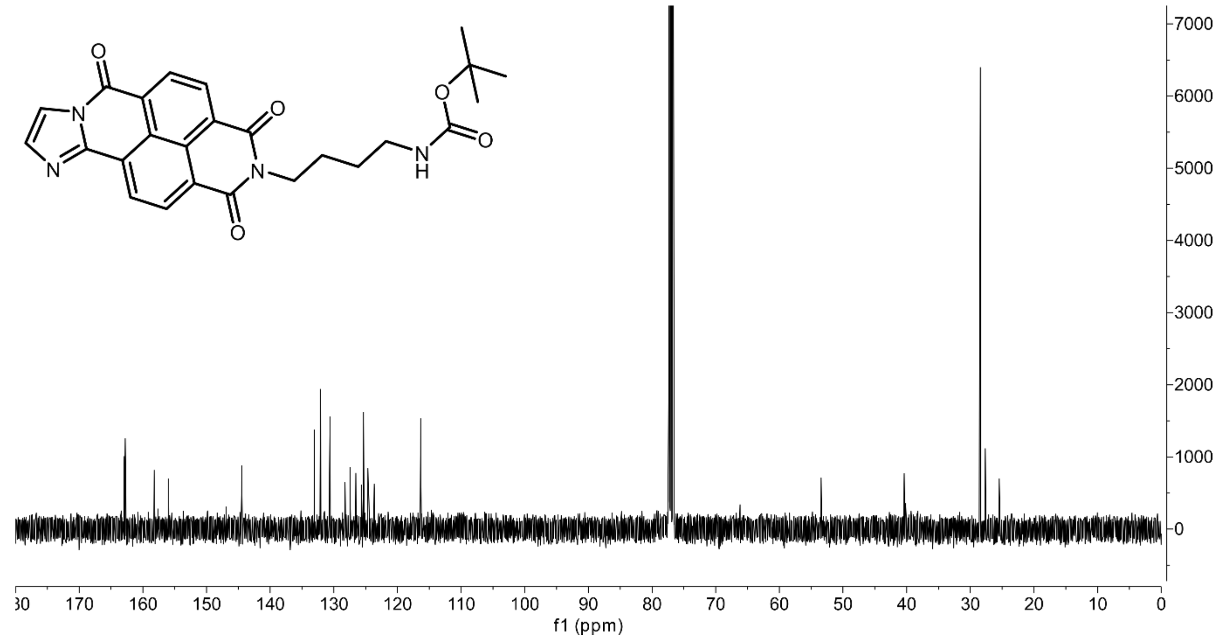

NMR 2: ^{13}C-NMR of NDI-DAE-Boc in $CDCl_3$.

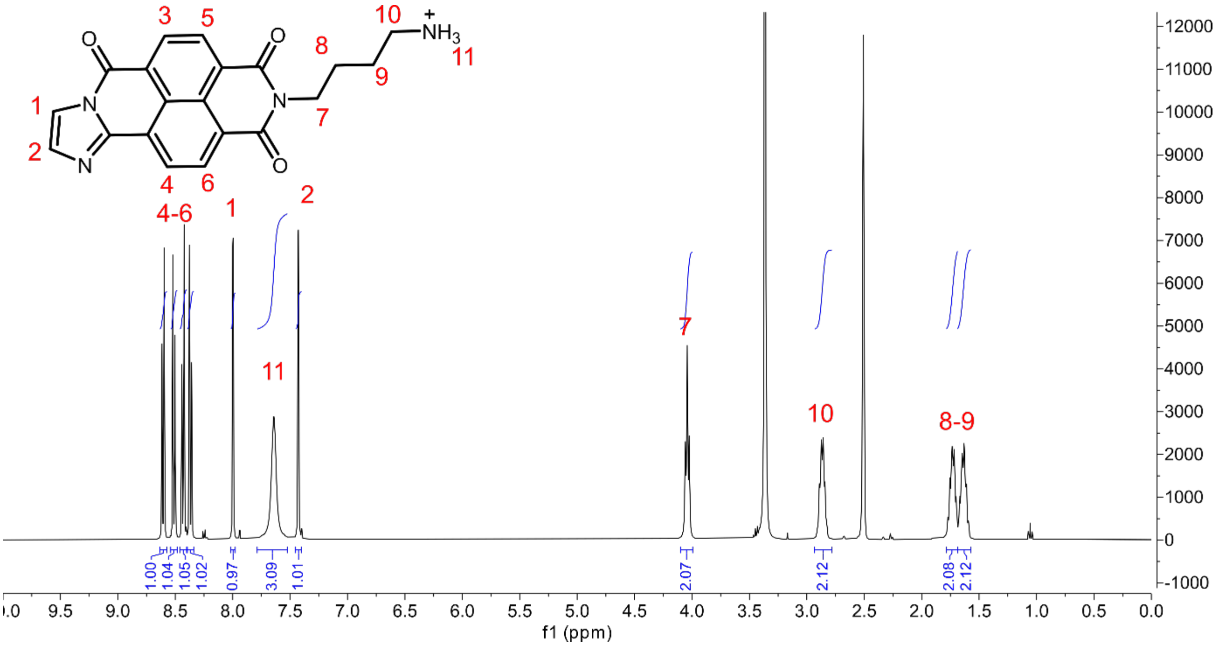

NMR 3: ^{1}H-NMR of NDI-DAE in DMSO-d6.

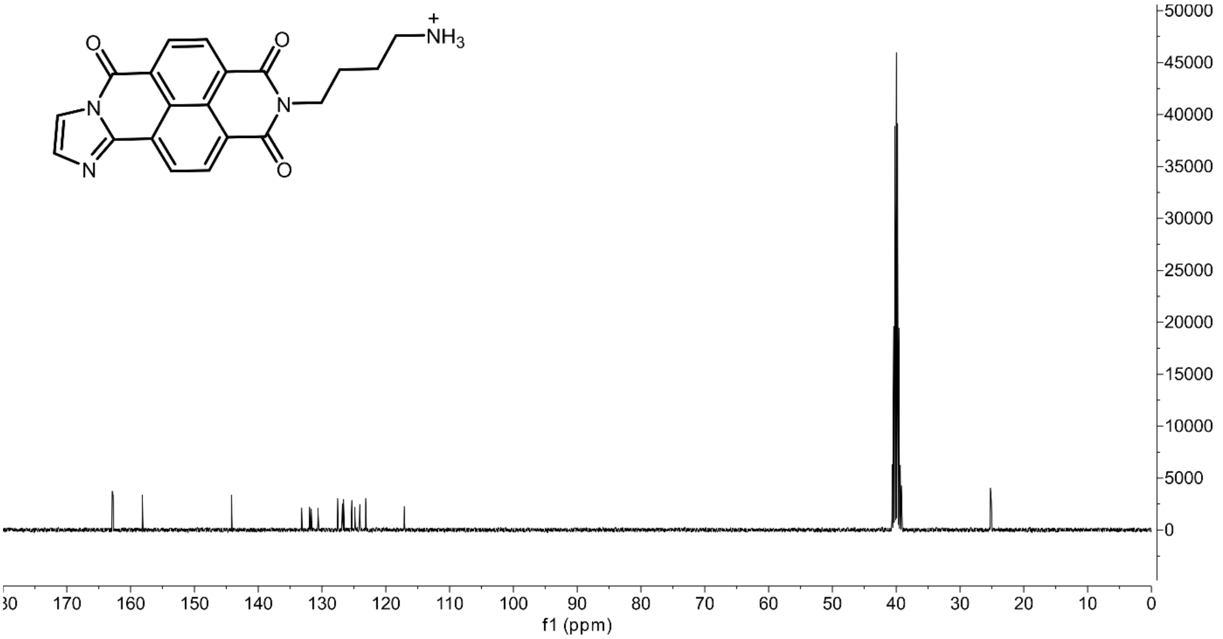

NMR 4: ^{13}C-NMR of NDI-DAE in DMSO-d6.

Supporting References

(1) Nussbaum, S.; Socie, E.; Yao, L.; Yum, J.-H.; Moser, J.-E.; Sivula, K. Tuning Napththalenediimide Cations for Incorporation into Ruddlesden–Popper-Type Hybrid Perovskites. *Chem. Mater.* **2022**, *34* (8), 3798–3805. https://doi.org/10.1021/acs.chemmater.2c00246.
